# Hypodermic Medication

**Published:** 1871-07

**Authors:** T. Curtis Smith

**Affiliations:** Middleport, Ohio


ARTICLE VII.—*Hypodermic Medication.* By T. CURTIS SMITH, M.D., Middleport, Ohio.

My excuse for this article is a desire to extend the knowledge and use of a very valuable means of treating many diseases. There is no method of treatment now in use as highly beneficial as this in painful and spasmodic affections, as well also as in many not of this class.

HISTORY.

Hypodermic medication was first discovered by Alexander Wood, of Edinburgh, in 1843. He was led to this discovery *First*, by being strongly impressed by Valleix' theory of there being four points in every sensitive nerve, more liable to be affected by neuralgia than any other portions.*

1st.—The point of emergence from its long encasement.

2nd.—When it traverses the muscle.

3rd.—Its peripheral termination.

4th.—When it approaches nearest the surface of the body.

The second step that led him to the discovery was the invention and use of an instrument to inject *nevi*, by Ferguson. Wood, believing neuralgia to be a local disease, it ought to be treated locally. He immediately devised an instrument to prove his theory, and had not long to wait before an opportunity offered to test it. "An old lady 80 years of age, the subject of cervico-bronchial neuralgia, was his first patient. An injection of thirty drops of vinous solution of morphia, on the shoulder at the sensitive point, gave almost instant relief, and soon put her to sleep. Next morning she was still asleep, but easily aroused. The one injection, although it caused at the time considerable alarm, entirely cured the patient of the neuralgia."†

After this, in and around Edinburgh, the hypodermic use of some preparation of opium was frequently practiced. Wood believed it necessary to localize the remedy to make it effectual for the relief of pain, though he recognized the systemic effects of the remedies thus applied. His first publication on the subject

* Gallaher, N. Y. Journal for May, 1871, pp. 532–3.

† Gallaher, N. Y. Jour. Med. May, 1871, p. 533.

was in 1855, twelve years after its discovery. His priority was disputed by* Rynd, of Dublin, for himself, and by the friends of Kurzak, of Vienna, for him. The theory of Wood and "the discovery of the practicability and utility of introducing medicine under the skin for the relief of local pain,"† and that it must be *locally applied*, constitutes the first period in the history of "Hypodermical Medication."

This theory remained undisputed until 1859, when Charles Hunter, of London, published his first paper, entitled "Experiments Relative to the Hypodermic Treatment of Disease."‡ He originated the term *hypodermic*, which is the one now generally used, though the term subcutaneous is perhaps equally proper. Hunter demonstrated "that the physiological and therapeutical effects of medicines thus administered are procured through the agency of the blood, and that § localization of the injection is not necessary."

Great praise is due to Wood for the discovery, and equally great to Hunter for his untiring efforts to extend the use of the remedy to the relief of a greater range of diseases than it could possibly have been under the localization theory of Wood. From 1855, and especially after Hunter's paper in 1859, the *hypodermic* method of administering remedies spread rapidly over Europe, and to this country. Courty and Behier, in France; Scanzoni, of Wurtzburg; Oppolzer, of Vienna; Graefe, of Berlin, and others, brought the remedy into use in their respective countries.

The first ‖ account I can find of its introduction into this country, *was in* 1858, by the late Prof. Geo. T. Elliott, of New York,¶ who introduced into Bellevue Hospital, the use of morphia hypodermically "in the treatment of sciatica." Since 1859, it has rapidly advanced in frequency of use, and the number who use it, but Austin states it to be "still very much unappreciated," and states further "there are still many practitioners who will not

* Bartholow, Hypo. Med. pp. 13–4.

† Bartholow, p. 13.

‡ Bartholow, Hyp. Med. p. 15.

§ Bartholow, p. 13.

‖ Bartholow believes it was first used by Ruppaner, of Boston, in 1860. See p. 18.

¶ R. B. Maurey, Amer. Jour. Med. Sci., Oct. 1866, p. 373.

admit that there *can* be any particular advantage in it which the old way of giving medicines does not offer."* Similar prejudices exist in this country. Those who employ the hypodermic method are largely in the minority. Some oppose it because it is an innovation upon an old method; others, still, unacquainted in a *practical* way with the mode of using it, are afraid of its power. Besides its first use by Elliott, we have in this country articles from Ruppaner,† Storer, and Warren, of Boston; Reeves, McElroy, and Bartholow, of Ohio; Catlin, of Connecticut; Dickson, Hutchinson, and Mitchell, of Philadelphia, besides others in various parts of the States.

### ADVANTAGES OF THIS METHOD.

There is perhaps no one who has given this method a fair and general trial, who will fail to speak well of its value as a means of treating many and various forms of diseases, and as being superior to the old stomachic method of administration in VERY MANY INSTANCES.

Gallaher, of Pittsburgh, Pa., states:‡ The hypodermic method of giving medicines is now so well established that no practitioner of any standing will denounce it, or refuse to avail himself of its advantages. Our periodicals, from every source, "are teeming with its triumphs," and men in the profession, high and low, throughout nearly every part of the world, are rapidly extending its usefulness, and the breadth and utility of its application in disease and for experimental purposes. Among its advantages may be named: 1st, and probably least, economy of medicine, which can only be an object in case of very high price, or great scarcity of an article. 2nd. Rapidity of action. The full effects of a remedy are manifested in from five to twenty minutes. This is a great object in very painful or severely spasmodic diseases, as colic, cholera morbus, neuralgia, congestive chills, convulsions, etc. 3rd. Intensity of effect. The intensity is greater, because the whole portion influences the nervous system, within a short space of time, compared with absorption by the stomach. 4th. The certainty of action. This is a *great object in any remedy.*

* Practitioner, July, 1868.

† Bartholow, p. 18.

‡ N. Y. Med. Jour. May, 1871, p. 535.

When a remedy is thus administered it is next to impossible for it to fail to act. It is placed in immediate contact with the absorbents and capillaries, and must, almost of necessity, be carried into the blood, which is the medium of its action on the nervous system. 5th. Greater permanence of effect. Instances are recorded of diseases neuralgic in character, having resisted the internal use of remedies for years, being permanently cured by a few hypodermic injections. Wood's first patient was entirely cured by one injection. One obstinate case of frontal headache, treated by myself, has remained cured for near two years, though but one injection was used. Another case of cephalalgia was relieved eight months by one injection, and eighteen months from a second. Of the permanence, Dr. Austin * says "the advantages of morphia, hypodermically administered, over the opiate medication by the stomach, are such as would be *a priori* incredible, nor can they yet be fully explained. In particular, it is impossible to account for the far greater permanence of its action in relieving nerve pain, which is so marked as that its discovery has initiated a new era in the treatment of neuralgias." 6th. Purity in the action of a drug. By this method a drug cannot be changed in character, as it often may be in the stomach or in its passage along the alimentary tube, by actions or reactions of the natural or morbid secretions with which they come in contact. No† such failure can occur in the therapeutic action when they are injected into the cellular tissue. 7th. The facility of affecting the system in cases where, from irritability of the stomach or rectum, the remedies cannot be retained, or, if retained, the morbid condition of the viscera prevents the absorption of the remedy, or the secretions to some extent neutralize it. 8th. Safety of the method. It is perhaps as safe as any method of administering remedies, when proper caution is used. Prof. Naussbaum, ‡ of Munich, was injected 2000 times, before any accident occurred, and this probably from injecting a vein, and not fatal. Gallaher mentions a physician in Pittsburgh, who has been operated on over 1400 times, without the occurrence of an unpleasant symptom beyond the production of two "pustules." Another under his care in

* Bartholow, pp. 53–4.

† Gallaher, N. Y. Med. Jour. May, 1871, p. 537.

‡ Gallaher, N. Y. Med. Jour. p. 536.

which he had used over one hundred injections without *any* unpleasant symptoms. Dr. Charles Hunter, in his remarks before the Clinical * Society, of London, reports an annual experience of over 2000 cases, without observing any risk in the method, " whether there was disease of the *heart* or not." Graefe has † injected the temporal region hundreds of times. Dr. Wood made over one hundred injections in one patient. Eulenberg used it in one case *four hundred times*, without meeting with a single instance of unpleasant results. I have myself used it several hundred times in different patients. *Thirty* times being the most in any one case, and have *never* noticed any serious effects from it yet. 9th. Saving of strength, or, as Hunter has it,‡ avoidance of exhaustion. This is an important advantage, for in cases of extreme pain, or spasm, the sooner it is cut short the better the chance for the patient. This may be instanced where the jaws are closed *spasmodically*, *preventing stomachic administration*, or when the *shock from the pain is so great that the system fails to respond quickly to remedies given by the mouth.* Cholera, colic, and tetanus, are examples of this. But these conditions do not prevent the rapid absorption and effects of the remedy *hypodermically administered.* Who of our readers has not seen cases which he knew he could snatch from the jaws of death if he could only get his remedies to act before the powers of nature gave way? In such cases, this is *the remedy with which to save*, *if not life*, *certainly a vast deal of suffering.* 10th. The advantage of the absence or avoidance of the unpleasant symptoms that often follow the stomachic administration of some medicines. This is certainly very marked in many cases, and Hunter argues that the avoidance is still more complete by inserting the remedy at a distance from the seat of pain. I have found many cases to whom I dared not give morphia by the stomach, but, nevertheless, complained very little from its hypodermic use. But as a rule, in my hands, the effects have been quite the same as by the stomach, but as I have usually practiced the use of the remedy locally, it may be, as Hunter charges, that this caused the ill effects of vertigo,

* London Lancet, March, 1871, p. 161.
† Amer. Jour. Med. Sci. April, 1866, p. 429.
‡ Am. Jour. Med. Sci. July, 1865, p. 207.

nausea, etc. 11th. Saving of time to the practitioner. This is often a great object when pressed with business or urgent calls. The difference between relieving the patient in five minutes by this method, and waiting from one-half to two or three hours for relief from pain by the old method, is frequently no small item of advantage.

There can be no question that the administration of remedies by the hypodermic method of introducing medicines into the subcutaneous cellular tissue is one of the most important additions to our remedial resources, that we have lately acquired.

As* a means of relieving pain, it rivals even the inhalation of chloroform in certainty, and rapidity of action, and surpasses it in permanency of effect, while it is comparatively free from objections which might be made in regard to dangers of fatality or slighter ill effects.

DANGERS.

Like other powerful means of relief, it will prove dangerous when improperly used. As in entering a vein, or in using too large a dose, from which sudden prostration, nausea, vomiting, excessive itching, painful abscess, or even death has been caused by its use. But these are never known to occur when due precaution is taken. *It is my opinion, based on careful observation, that the injection of a vein can quite invariably be avoided by inserting the needle one inch under the integument into the cellular tissue, and then before injecting the solution,* WITHDRAW THE POINT OF THE NEEDLE ONE-FOURTH OR ONE-HALF OF AN INCH, *then raise the integument with the needle, holding the instrument parallel with the surface into which it is inserted. By this precaution, the vein if punctured will be pouring out its own blood, and will, therefore, not be able, by any possibility, to carry the solution directly to the heart. The only exception I can conceive to the success of this variation would be when the needle happened to traverse the course of a vein for the last quarter or half inch of its course* UNDER *the skin.* CERTAINLY A RARE OCCURRENCE. *But even if a vein should thus be entered, the little force employed to elevate the integument with the needle, would rupture the thin walls of so small a vein, thus allowing it*

* Amer. Journal Medical Science, April, 1866, p. 427.

*to pour out its own blood into the cellular tissue, and while this is taking place, the surrounding* CAPILLARIES *and* ABSORBENTS *will take up the solution, leaving the poured-out blood the* LAST TO BE TAKEN UP. *I have never seen this direction to avoid injecting a vein given by any authority,* BUT CONSIDER IT OF VITAL IMPORTANCE THAT THIS PRECAUTION BE STRICTLY OBSERVED.

Professor Naussbaum,* after injecting himself over 2000 times, injected the medicine (2½ *grs. morphia,*) into a subcutaneous vein. This was followed by very great prostration, from which he recovered. For myself, I have never seen any symptom more unpleasant than that of occasional nausea, vomiting, vertigo, and itching of the skin and nose. All of these, except the latter, are quickly relieved by bromide potassium, given by the stomach, or injection.

Morphia is, perhaps, more fruitful of these ill effects than any other remedy, it having a physiological tendency to produce them, and being used more frequently than all other remedies combined.

Drs. Southey and Duckworth† each relate a fatal result. The former from 1-6 grain of Sulph. Morphia, in a case where the heart was seriously diseased. Dr. Hanfield Jones‡ relates in brief, three cases in which the remedy was followed by serious fainting. In these he thought there was serious disease of the valves or muscular tissues of the heart. Gallaher§ mentions a case in his experience, where severe prostration was produced, lasting twenty-four hours. In nearly all of these cases a vein seems to have been entered.

### PHYSIOLOGY.

Remedies admitted subcutaneously have quite the same physiological effects as when given by the mouth. Their effects, however, are *more powerful* in the same *sized doses in the former than in the latter*, and in some instances differ in kind.

A scientific ‖ committee appointed by the Clinical Society of London, to report on the physiological and therapeutical effects of remedies administered by this method, gave it as their opinion,

* Am. Jour. Med. Science, June, 1866, p. 240.

† London Lancet for March, 1871, pp. 161-2.

‡ Ibid.

§ N. Y. Med. Jour. May, 1871, p. 537.

‖ American Jour. Med. Science, Oct. 1867, p. 537.

that no difference has been observed in the effects of a remedy *thus given*, and by their *introduction into the stomach, except greater rapidity, certainty and intensity of effect, and requiring a less amount to affect the system than when given by the stomach.* There were, however, some exceptions to this rule. When quinia was injected into the cellular tissue, considerable elevation of temperature was observed. This symptom being slight or inappreciable when the drug was taken (in the same quantity) by the mouth or rectum. "A solution of podophyllin injected under the skin gave rise to free diuresis, a symptom which was characteristic of this method of administering the drug."

I have occasionally noticed nausea, and in nearly every case some degree of constipation produced by morphia. This latter is probably caused by the sedative action of the drug on the nervous system, as it would be impossible for it to have any astringent power over the mucous lining of the alimentary canal, when given hypodermically. There are, however, many statements to be found from good authority, to the contrary of this opinion.

### THERAPY.

Remedies introduced into the system by the stomach, are often affected by the condition of that organ, or those organs annexed to it, as by inflammation, congestion, repletion of the veins, ingesta and character of the gastric juice. The condition of the liver, spleen and pancreas also produce some effect upon the action of the drug. The same may be said of the condition of the mucous membrane of the alimentary tube beyond the stomach. These conditions may be such as to prevent the absorption of a remedy, or to so far alter its character by chemical action, as to entirely change its therapeutic action on the system. I have many times found the stomach in such a morbid state as to fail to absorb either *morphia or opium.* The same may be said of other drugs. Besides, the use of remedies of the narcotic class may so far depress the nerves of the stomach, as to greatly limit the rapidity or extent of absorption, thus limiting the power of the remedy, or rendering its absorption so slow, as to quite prevent any noticeable effect. On* the other hand, when a medicine suitable for this purpose is thrown under the skin, its physiological and therapeutical effects

* Bartholow, p. 29.

are produced in the fullest degree, and in the most characteristic form. Bartholow says: "It follows then, that the therapeutical powers of a drug must not only differ in degree, but also in kind, according as it is given by the stomach, or injected under the skin. The subcutaneous use of certain drugs has developed very valuable therapeutical properties, which the stomachic administration had not even suggested. This is demonstrated by the effects of podophyllin,* and other drugs given *subcutaneously*. The *advantages* of this method have been treated of before. (See *ante.*) But I shall again ask attention to one point, the seventh in the list, viz., The facility of affecting the system in cases where from a morbid condition of the stomach or rectum, or from the power of absorption being checked by severe shock from injury or very *extreme* pain, the remedy fails to act, when given by the usual method. We have all seen such cases. I will relate two in brief, from among others that might be added.

I was called hastily one morning to see a gentleman who was suffering from an extreme attack of cholera morbus. Not having any syringe, I used autacids, opium, morphia, hot drinks, stimulating counter-irritants, heat, etc. Being obliged to leave to see others, these were ordered to be continued till relief was afforded. Two hours later a messenger announced that the man was no better, and that he was sinking rapidly, and would die if not relieved. I assured him that the man should be relieved in a few minutes. On reaching the house I found the man cramping from head to foot, pain excruciating, could perceive no pulse at the wrist, and the extremities were cold, surface bathed in a profuse cold sweat. I immediately injected a half grain of morphia sulph. In ten minutes the effects of relief were very apparent, in twenty minutes the man was easy and asleep. The vomiting and purging immediately ceased. This case, in the times of epidemic cholera, would unhesitatingly have been pronounced an instance of that fearful disease.

Case 2.—I was called last summer to see a lady, who was suffering from very extreme ovarian neuralgia. She was not one who complained for nothing. I think she was in as much

* Of 30 or more injections of podophyllin which were administered, only one produced any morbid diuretic effect, and that was, probably, a mere coincident.

agony of pain as any one I ever saw. I used opium and quinine largely; then morphia, chloroform, heat, stimulants and counter-irritants, for three hours, without the least appreciable effects. Prostration seemed to be approaching. I sent for the hypodermic syringe, and gave 15 M.'s Magendie's Solution Morphia. In 15 minutes she was much better, but still suffering. I then gave 10 M.'s more, and in five minutes she was quite easy and sleeping. I should have stated that morphia was administered per rectum to the extent of one grain, but no effect seemed to be derived from it. Fearing narcotic poisoning from the large quantity of opium in the *stomach*, I gave an emetic of zinci sulphas, and a very large injection of warm water, per rectum, which latter moved the bowels freely, and the former producing free emesis. As I expected, the opium quite unchanged, mixed with an abundance of glairy mucus, was thrown off in the act of vomiting. This lady was not an opium eater.

Other cases could be named, but it is needless to do so; these quite representing the very great advantages of the hypodermic method. I seldom go about now without my hypodermic instrument with me, having as I believe lost one case at quite a distance from home, for want of it.

The committee appointed by the Clinical Society of London, state, "That* no difference has been observed in the effects of a drug subcutaneously injected, whether it be introduced at a distance or near to the part affected." Others, high in authority, express the same opinion. From this view it may seem absurd for one occupying a position as humble as I do, to dissent. But in a certain class of cases where the pain has been *long* and *immovably seated in one location*, *the remedy locally applied is certainly more effectual* than when administered at some distant part. Drs. Ruppaner, of New York; Eulenberg, of Berlin; Behier and Lorent, of France; Gallaher, of Pittsburg, and especially, Wood, of Edinburg, maintain that localization *is often followed by better and more permanent relief than when injected at a distance.* Eulenberg states that in a case† of double rheumatic sciatica, observed by him, *complete relief* of pain, for a space of from two to three

* Am. Jour. Med. Science, Oct., 1867, p. 531.

† American Journal Med. Science, April, 1866, p. 432.

days, followed each injection *upon the side upon which the injection was made.* While upon the other side, the pain *immediately returned*, upon the subsidence of the effect of the narcotic upon the nervous centres.

The reasons for localization are: 1st. The injection influences the nerves of the part injected, producing a partial local anæsthetic effect of considerable permanence, sometimes lasting for several days. 2d. The local shock attending the operation tends to break up the established habit of local morbid influence. 3d. In addition to the local influence, the part receives also its share of the systemic effects, thus giving the one locality a double effect, and hence more power over the local disease. I have already cited two cases in which the remedy, applied locally, had a most happy effect, while I had no reason to expect permanent good from it if injected at a distance, (see *ante*) (two cases head pain.)

The diseases in which this remedy has been beneficially used are numerous. Among them we may especially place those severely painful and spasmodic, as well as many that are of cerebral origin, as mania, melancholia, delirium tremens, etc. And of the digestive organs, as sea sickness, excessive vomiting, colic, intussusception, etc. These should be treated under the head of Special Therapeutics, but space and time will not allow more than a brief notice of a few diseases, after which I shall describe the instrument, manner of using, and formulæ for some of the leading remedies used by this method, then close this already lengthy article. I will give a very few of the many cases in which I have used the hypodermic method of treatment.

Case 1.—On the 6th day of August, 1869, I was called to see Mr. Mc., who was suffering from a severe attack of pleurodynia. I injected, over the pain, six minims Magendie's Solution. In five minutes he was relieved without a bad symptom, though he could not move or even breathe without severe pain.

Case 2.—Was called on the night of Sept. 1st, 1869, to see Mrs. T., suffering severely from cholera morbus. Everything administered, even water, was immediately rejected. I administered one-sixth of a grain of morphia, under the skin. In ten minutes the pain and vomiting all ceased, and she was cured without a bad sequence.

Case 3.—Mr. M. who frequently had attacks of colic, as well also as his wife, always requests its use, in preference to waiting for the old slow plan of treatment. It has generally relieved them in from eight to ten minutes.

CASE 4.—Mrs. R. was awakened one night by a terrible pain in the shoulder, caused by muscular spasm; she could not move without great pain, and her suffering seemed very great, even while lying still. I sent one and one-half grains of opium, which she took, but without relief. I *saw* her some hours later, still suffering, and injected over the deltoid muscle ¼ gr. sulph. morphia. After 10 minutes she had no more pain.

CASE 5.—Mr. E., the subject of a knot of varicose veins on his hip, applied for relief from this ugly deformity. I injected the veins as full as possible of a saturated solution of ferri persulph. This enlarged the veins and rendered the little tumor quite sore for a few days, after which the difficulty disappeared.

CASE 6.—Mr. D. was taken with a most agonizing pain over the region of the left kidney, and down the course of the ureters. He was immediately prostrated; extremities cold. Pulse very feeble; respiration labored. It was just such a case* as one would naturally expect opium to be hours in relieving, if given by the stomach, even in conjunction with other remedies. I at once injected 15 minims of Magendie's Sol. In 15 minutes the pain was very much abated, but still severe. I gave 10 minims more, and in 10 minutes he was perfectly easy, but greatly prostrated from the effects of the pain. The morphia produced no ill effects.

These cases could be multiplied, but it is needless. I will turn to a few cases given by others.

Dr. W. Haining reports a case of tetanus cured by injections of Calabar bean.† Dr. Fraser reports the cure of nine cases out of eleven‡ by the same remedy, but he combined both methods of administration.

Strychnia has been successfully used to relieve paralysis, and as a tonic, after it has failed to produce the desired effect by the stomach. Hunter cured§ one case of six years', and another of

* Probably a case of the passage of nephritic calculi.

† London Lancet, March, 1870.

‡ N. Y. Jour. Med., May, 1871, p. 538.

§ Bartholow Hyp. Med., p. 104.

two and one-half years' standing; another case, time of duration not stated. He and Echeveria have treated all forms of paralysis by this method successfully. The latter recommends great care in discriminating the proper cases for its use in this disease. It should not be used by this method in cases where it is contra-indicated by the stomach. Morphia, and its salts, are the most important of the whole list for this method. The sulphate, being the most soluble, is the one commonly used; the ordinary dose is from one-eighth to one-half grain. The dose should be small at first, until we know how each case will bear it. Doses of larger size may be given in extreme cases of pain. The morphia habit may be established by this method, the same as by the stomach. Dr. Buzzard* reported a case to the "Clinical Society of London," of a lady who had frequently used thirty-three grains of morphia in twenty-four hours, by subcutaneous injection. He injected fifteen grains at one dose without bad effect. First in the list for relief by morphia stands neuralgia, in all its forms and locations. It is often cured by a few injections, after resisting other treatment for months. In delirium tremens it is highly extolled by nearly every authority. The danger of narcotic poisoning is less, for the reason that each portion is immediately taken up, and its effects produced to procure sleep. In hysteria it is one of our most reliable medicines. I have used it with the happiest effects. Hunter, Lauder, Frovmiller and Lorent speak well of it. Bartholow says:† "In my own experience, no remedy has acted so promptly and satisfactorily in terminating a hysterical paroxysm as this." In puerperal mania it is highly commended, and those who have used it consider it safer and far superior to chloroform. In all diseases having their origin in morbid reflex-excitability it is perhaps the best remedy at our command. In tetanus, it is rapidly coming into use, and should certainly be tried in every case where stomachic administration is difficult. In local or general muscular cramp, it cannot be too highly estimated. In spasmodic asthma, there is perhaps no means we can command that will compare with the subcutaneous administration of morphia or atropia, or the two combined, in *rapid* and *complete* relief. I have

* London Lancet, March, 1871, p. 161.

† Bartholow, p. 49.

seen almost perfect relief follow in five minutes. Atropia is probably the best of the two remedies, but *the two combined is better still.* In hiccough, angina pectoris, pleuritis, etc., it is a certain and valuable remedy. In *cholera*, Bartholow says: "The most instantaneous and striking relief is afforded by the hypodermic injection of morphia." One of the most important effects is in the relief of congestive chills. Dr. Jos. E. Garrison,* Asst. Surg. U. S. A., "reports five cases in which morphia, injected subcutaneously, absolutely cut the paroxysm in a *few minutes*," and the subjects were able to sit up and walk about in a remarkably short space of time. One, though so far depressed as not to know of his presence, so far recovered *before he could put up three* five grain powders of quinia, that she asked to get out of bed, and did so. Morphia, by this method, is highly extolled for the relief of pain in cancer, and Bartholow asserts that "in scirrhus of any portion of the digestive tract, especially of the stomach, no palliative is comparable to the hypodermic injection of morphia."†

The physiological antagonism between morphia and atropia is now pretty well established. Poisoning by the one may be readily antagonized or antidoted by the other. A very interesting array of facts and cases may be found in the Medical and Surgical Reporter, April 22, 1871, p. 334, by Dr. John A. Little, of Delaware, Ohio, in which he gives his own experiences, and the authority of standard teachers in support of this fact. The report will repay perusal. The therapeutic applications of atropia are as important as those of morphia, but the number of diseases in which it has, or may be used, are not so great. In asthma, it is very highly extolled, but I can only give it a passing notice. *Atropia, combined with morphia, will very often obviate the ill effects of the latter, without destroying its anodyne effect, while at the same time it really increases the hypnotic power of the morphia.*

The uses of quinia, by this method, are very valuable and effectual. Dr. Moore, of Bombay Medical service, states‡ that four or five grains of quinia, injected beneath the integument, are

* Amer. Jour. Med. Science, April, 1870, p. 571.

† Bartholow, p 64.

‡ Bartholow, p. 131.

equal to five or six times that amount taken into the stomach. Not only are its effects immediate, but greater and more permanent, being less liable to be followed by a relapse. Bartholow states that from three to five grains are sufficient "to cure an ordinary intermittent." In* those deadly malarial attacks, known as "puniceous," is the efficacy of this treatment most conspicuous.

We all know the importance in these cases of bringing the patient rapidly under the influence of the remedy, hence the great advantage of this over other methods of treatment. It is my opinion that a combination of morphia and quinine is more appropriate, and would be more effectual than either one alone.

Dr. R. B. Maurey, of Port Gibson, Miss., gives a report of twenty-five cases of the various forms of malarious fevers treated with quinia by the hypodermic method. He states that "in most of the cases six grains was the entire quantity of quinia used during an intermission or remission; in the severer cases eight grains was the quantity used. Cinchonism was fully produced in from forty to sixty minutes." He thinks that life can often be saved by this method in congestive intermittents, when it could not be done by the stomachic method, for the reason that absorption by the stomach is too slow in cases where the chill is near or already at hand, or that it may be so irritable as not to retain the remedy long enough to affect the system.

Many other remedies appropriate for this method have been very successfully used, such as conia, caffein, ergotin, mercury, arsenic, physostigma, etc. Of these I cannot speak for want of space, further than to call attention to the latter as an excellent remedy in tetanus, for which it is considered by many our best remedy. *But especially would I ask attention to the fact of its being the antidote for strychnia poisoning.* It is† the true physiological antagonist of strychnia. While strychnia exalts, physostigma destroys the reflex faculty of the cord. The rapidity, certainty and intensity of effect by this method render it valuable in both of these difficulties, but caution should be observed not to use too large doses, one-third of a grain of the extract being sufficient to commence with.

Different instruments are used for this method, but all are quite

* Am. Jour. Med. Sci. Oct. 1866, pp. 671–3.

† Bartholow, p. 124.

similar in construction. They are made of glass, gutta percha and silver. The glass instruments are graduated on the barrel—the others are graduated on the piston-rod—thus enabling us to know just how much of any solution we have in the syringe, by knowing the strength of which we can give precisely the dose desired. If air is drawn into the syringe it is inserted and the piston pushed in till it is all excluded. The syringe charged and ready for use, the integument is pinched up, between the thumb and finger, the point of the needle placed against the skin at the place where it is designed to enter. It is then passed through the skin by a *quick thrust*, after which it is carried about an inch into the cellular tissue by a slow movement, then slightly withdrawn, and skin raised, (see *ante*), always being very careful to avoid any vessel or nerve, especially a vein. The fluid is then discharged into the cellular tissue *very slowly*, *drop by drop*, till the requisite amount is discharged. I always deem it better to have a little more in the syringe than is injected. This makes it doubly sure against the introduction of air. Some authorities recommend that the tube be left a few minutes, so that if a vein has been entered, or any ill effects are likely to be produced, the barrel can be again screwed on to the tube and much of the fluid withdrawn, thus preventing the absorption of the whole portion administered.

The best situation for injections are the outer surface of the arm, the deltoid surfaces, the abdomen, back, chest, and outer face of the arms and thigh.

The effects are produced with about equal rapidity from each of these sites. Bony prominences and vascular sections should be carefully avoided.

It is better not to inject an inflamed surface, nor to inject *twice precisely in the same place*, as abscess or pustule might result from a neglect of this precaution.

In cases of long seated, obstinate pain, it is better, in my opinion, ceteris paribus, to inject at or very near the painful point, for reasons before stated. (See *ante*).

SOLUTIONS.

In this method much depends on the purity of the medicines, and the character of the solutions used. *All medicines used*

*should be in a perfect state of solution*, as particles of undissolved medicine, when introduced into the subcutaneous *cellular tissue*, are not readily absorbed, and are liable to act like a foreign substance, producing inflammation and abscess; besides, the foreign substances block np the cavity of the needle. It is always better to filter carefully the solutions, thus removing all dirt and foreign matter or undissolved particles of medicine.

The solutions should be as nearly *neutral* as possible, and *not too greatly concentrated nor acrid.* Any solution capable of coagulating the circulating fluid, or any of its constituents, should be avoided, except in NEVI and VARIX.

Pure distilled water is best for preparing solutions. *This* can be injected in any reasonable quantity without harm. It is better to court quantity to a reasonable extent, than too great concentration, for it is not so much the *quantity* as the *strength* that causes ill effects. Solutions, especially of the alkaloids, should not be too long kept, for the reason that PENICILLI* develop in them at the expense of the alkaloid, thus rendering the solutions of uncertain strength, and not so safe for use.

FORMULA.

Probably the best formula for morphia is Magendie's† Solution, which consists of: ℞ Morphia Sulph. grs. xvi; Aqua Destill., ℥ i; M. Of this, 30 minims equals *one grain*, the usual dose being 5 to 8 minims. For morphia and atropia combined, the solution must vary according to the desired purpose. As one *grain* of morphia *is equal in toxic power to* 1-24 *grain atropia*, from this we can gauge the dose. A very good formula is:‡ ℞ Morphia Sulph. grs. xvi; Atropia Sulph. gr. i; Aqua Destill., ℥ i; M, and filter, 5 minims the average dose. More morphia and less atropia can be used, or *vice versa*, as we desire to affect the patient. *The latter increases the hypnotic effect of the morphia.*

For atropia a very good formula is: ℞ Atropia Sulph. grs. ss; Aqua Destill., ʒ iii; M. Of this, 4 minims equal 1-90 of a grain. The strength *can* be doubled, but it is better not to do so.

Of strychnia a proper solution is: ℞ Strychnia Sulph. grs. ii;

* Bartholow, p. 20.

† Bartholow claims it as his own; who is correct?

‡ Bartholow, p. 89.

Aqua Dest. ℥ vi; M. Of this, 5 minims equal 1-36 of a grain, probably the average dose by this method, but it would be better to use only 1-50 of a grain at first.

Of quinia sulph. a proper solution is: ℞ Quinia Sulph. grs. xx; Acid Sulph. Arom. gtt. xx; Aqua Dest. ℥ iii; M, and filter. Of this, 9 minims equal 1 grain.

It is to be regretted that quinia is not readily soluble, without the aid of considerable acid; but by careful management ill effects can be avoided. I have injected two grains with good effect, and without bad after consequences. Of solutions for other remedies, I have not space to speak; however, I think it proper to add that this method, though possessing a very wide range of application in many and various diseases, should not so far be used as to exclude the use of remedies by the usual method in most diseases, where they are of long continuance. But as it is yet in its infancy, we are hardly able to predict whether or not in course of time it will take largely the place of stomachic administration. A report is already in print of its being very highly successful, by the injection of *calomel* in constitutional syphilis.* But if it could only be used for the relief of very severe pain, and in severely spasmodic diseases, it should be understood and used by every practitioner of medicine and surgery.

Since writing this article I have received an account from Dr. Thomas E. Pickett, of Maysville, Ky., of a man who by mistake received and took at once eight grains of sulph. morphia. Its poisonous effects were very soon manifested. All the usual means were immediately resorted to for his relief, viz.: flagellation, forced exercise, counter-irritants, cold douche, titillation, slapping the soles, galvanism, etc. The jaws were closed by tonic spasm of the muscles, preventing all attempts at relief by evacuating the stomach, or introducing any remedy whatever into it. All these means failed, and the patient's condition became desperate and quite hopeless. Respirations, 3; pulse, 140; extremities cold, etc. Atropia was now hypodermically used, at the rate of 1-24 grain every thirty minutes. After the fourth dose, improvement occurred, relaxation of the muscles allowed the jaws to open, which was taken advantage of for the use of stimulants by the stomach pump. The patient made a good recovery. The dose

* Lancet and Observer, May, 1871, from Lancet—Med. Gazette.

had been swallowed five hours or more before the atropia was used. The dose of the latter as an antidote in this case was remarkably small, yet its effects seem to have been all that was desirable. Larger doses would have afforded more prompt relief.

